# Polyphenolic Composition of *Crataegus monogyna* Jacq.: From Chemistry to Medical Applications

**DOI:** 10.3390/nu7095361

**Published:** 2015-09-11

**Authors:** Seyed Fazel Nabavi, Solomon Habtemariam, Touqeer Ahmed, Antoni Sureda, Maria Daglia, Eduardo Sobarzo-Sánchez, Seyed Mohammad Nabavi

**Affiliations:** 1 Applied Biotechnology Research Center, Baqiyatallah University of Medical Sciences, Tehran 1193653471, Iran; E-Mail: Nabavisf@gmail.com; 2 Pharmacognosy Research Laboratories, Medway School of Science, University of Greenwich, Chatham-Maritime, Kent ME4 4TB, UK; E-Mail: S.Habtemariam@greenwich.ac.uk; 3 Neurobiology Laboratory, Atta-ur-Rahman School of Applied Biosciences, National University of Sciences and Technology, Sector H-12, Islamabad 44000, Pakistan; E-Mail: touqeer.ahmed@asab.nust.edu.pk; 4 Research Group on Community Nutrition and Oxidative Stress, University of Balearic Islands, and CIBEROBN (Physiopathology of Obesity and Nutrition), Palma de Mallorca E-07122, Spain; E-Mail: tosugo@hotmail.com; 5 Department of Drug Sciences, Medicinal Chemistry and Pharmaceutical Technology Section, University of Pavia, Via Taramelli 12, Pavia 27100, Italy; 6 Laboratory of Pharmaceutical Chemistry, Department of Organic Chemistry, Faculty of Pharmacy, University of Santiago de Compostela, Galicia 15782, Spain; E-Mail: e.sobarzo@usc.es

**Keywords:** *Crataegus monogyna*, hawthorn, catechins, flavones, pharmacological activities

## Abstract

The abundance of scientific evidence has shown that many synthetic drugs can cause serious adverse effects in patients. Recently, the search of natural therapeutic agents with low adverse effects has attracted much attention. In particular, considerable interest has focused on edible and medicinal plants, which play an important role in human diet, and have been used for disease treatment since ancient times. *Crataegus monogyna* Jacq. (hawthorn) is one of the most important edible plants of the Rosaceae family and is also used in traditional medicine. Growing evidence has shown that this plant has various interesting physiological and pharmacological activities due to the presence of different bioactive natural compounds. In addition, scientific evidence suggests that the toxicity of hawthorn is negligible. Therefore, the aim of this paper is to provide a critical review of the available scientific literature about pharmacological activities as well as botanical aspects, phytochemistry and clinical impacts of *C. monogyna*.

## 1. Introduction

Natural products have been of great importance in disease treatment since ancient times. In fact, in traditional medicine, medicinal plants and herbal formulations play a crucial role in the prevention and mitigation of different human diseases. During the past two decades, herbal medicines have received considerable attention as novel therapeutic options for human disease treatment [1,2,3,4,5]. It is widely accepted that the presence of different bioactive compounds is responsible for the pharmacological effects of medicinal plants, among which edible plants are the most promising, due to their negligible adverse effects [6,7,8].

**Figure 1:**
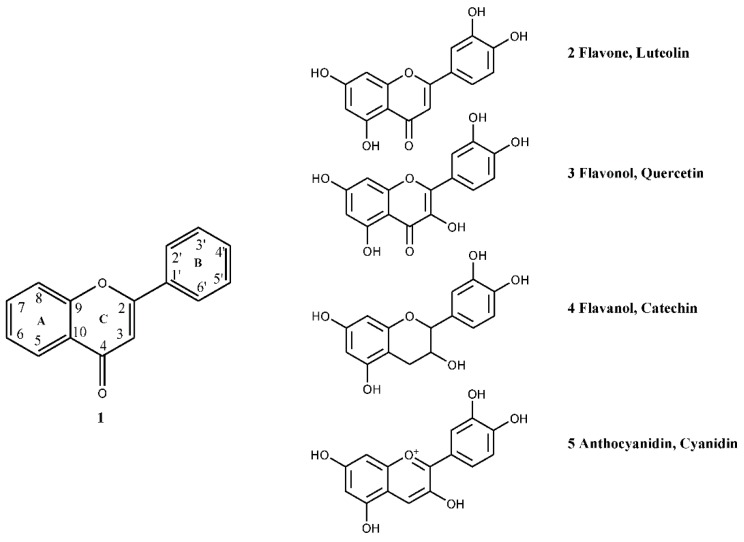
The classes of *C. monogyna* flavonoids.

*Crataegus monogyna* Jacq. (common hawthorn) is an endemic member of the Rosaceae family that grows in Europe, Africa, and Asia, where is commonly found as a shrub or small tree 5–10 m tall [9]. Its small dark-red fruit (commonly called haw), which ripens in mid-autumn, is used for different culinary purposes, such as the preparation of jellies, jams, and syrups [10]. Scientific evidence has demonstrated that hawthorn fruit possesses potent antioxidant and free radical scavenging activities, due to the presence of different bioactive compounds, such as epicatechin, hyperoside, and chlorogenic acid (Figure 1) [11,12]. These compounds are reported to have many pharmacological effects, including neuroprotective, hepatoprotective, cardioprotective, nephroprotective, *etc.* [13,14]. Furthermore, hawthorn fruit possesses tonic effects on the heart [13]; several studies have shown that it could reduce some cardiovascular risk factors, such as hypertension, hypercholesterolaemia, *etc.* [9,15,16]. Despite this growing body of evidence, to date there has been little attempt towards a coherent understanding of the potential health effects of hawthorn. Therefore, the aim of this paper is to provide a critical review of the available literature, regarding traditional use, chemical composition, biological, pharmacological, and toxicological effects of hawthorn.

## 2. Methods

This study consists of an up-to-date review of the literature, which contains data about chemical composition, pharmacological studies, and medical applications of *C. monogyna.* Criteria for selecting the material were as follows: a search was conducted on the PubMed database [17], using the keywords “*Crataegus monogyna*”. The results returned 88 papers up to 2015; these were summarized and critically discussed to provide a consistent review. A second search was conducted on the ClinicalTrials.gov database [18], using the keywords “hawthorn” and “*Crataegus monogyna*”; this returned 23 clinical trials on this plant, which are summarized in Table 1.

In the following sections traditional uses, phytochemistry, biological and pharmacological effects, adverse effects, drug interaction and clinical impact of *C. monogyna* will be discussed.

**Table 1:** Details of completed clinical trials according to our search with keywords “hawthorn” and “*Crataegus monogyna*”.

NCT Number	Study Type	Conditions	Study
NCT01331486	Interventional	Prehypertension; Mild Hypertension	Nitric Oxide Mediated Vasodilatory Response to Hawthorn Standardized Extract
NCT00794456	Interventional	Anxiety Disorder	Association of *Passiflora Incarnata* L; *Crataegus Oxyacantha* L and *Salix Alba* L. on Mild and Moderate Anxiety
NCT00006330	Interventional	Heart Diseases	Pharmacokinetic and Pharmacodynamic Interaction Study of Digoxin and Hawthorn
NCT00343902	Interventional	Chronic Heart Failure	Hawthorn Extract Randomized Blinded Chronic Heart Failure (HERB CHF) Trial
NCT01482819	Interventional	Myopia	Evaluation of Daytime Corneal Swelling During Wear of Galyfilcon A Lenses
NCT00455026	Interventional	Depth of Anesthesia	Effect of Remifentanil on Electroencephalographic BAR Index During Propofol Anesthesia
NCT00226837	Interventional	Depth of Anesthesia	Quantifying Nitrous Oxide Effect on Depth of Anesthesia Using Theoretically Based Time Series Modelling
NCT01444287	Interventional	Myopia	Daytime Corneal Swelling During Wear of Narafilcon B Lenses
NCT00762502	Interventional	Astigmatism	Comparison of Senofilcon A Toric Lenses to Balafilcon A Toric Lenses Over
			Extended Wear Period
NCT00027352	Interventional	HIV Infections	A Comparison of Two Ways to Manage Anti-HIV Treatment (The SMART Study)

## 3. Traditional Uses of *C. Monogyna*

In both Europe and China, hawthorn fruit is commonly used for preparation of many foodstuffs, such as jam, jelly, drink, and wine [9]. In traditional medicine, hawthorn has been widely used to treat human diseases [11,14]. Its medical properties were first described by Dioscorides in *De Materia Medica*, first century A. D., which formed the core of the European pre-modern pharmacopoeia. In Traditional Chinese Medicine (TMC), hawthorn was mentioned in the first state-approved pharmacopoeia: the *Tang Ben Cao*, 659 A.D. In Europe the most common species used for medicinal purposes are *C. monogyna* and *Crataegus laevigata* (Poir.) DC. (which is the accepted name of *Crataegus oxyacantha*), while in China, *Crataegus cuneata* Siebold & Zucc. and *Crataegus pinnatiftida* Bunge are the most well-known and used species [9,19]. In folk medicine, hawthorn has been used for the treatment of cardiac diseases, hypertension, hyperlipidemia, and as anti-atherosclerotic agent [15,20,21]. It has been reported to be especially effective against cardiovascular problems, such as heart failure, hypertension with myocardial injuries, angina pectoris, arrhythmia, and atherosclerosis. In addition, it has been used for improving blood circulation system, as well as blood stasis elimination [15]. Hawthorn has also been used for the treatment of gastrointestinal diseases, stimulation of digestion, and promotion of stomach functions. Moreover, hawthorn had application in the treatment of indigestion, epigastric distension, abdominal pain, and diarrhea. In the European tradition, hawthorn is also used as an anti-spasmodic, cardiotonic, astringent, and diuretic agent [22,23,24].

## 4. Phytochemistry of *C. Monogyna*

In view of the traditional medicinal uses of *C. monogyna*, modern scientists have extensively investigated the chemical constituents, to which the pharmacological effects could be attributed. The secondary metabolites, extracted from the different parts of the plant, range from simple fatty acids to terpenoid and polyphenolic compounds. Among these latter, many polyphenols were detected in *C*. *monogyna*, especially in the plants grown in Chile [25]. Several compounds possess antioxidant properties; these include chlorogenic acid, epicatechin, hyperoside, quercetin, rutin, vitexin, and procyanidins [26,27,28]. Generally, flavonoids, particularly flavonols and flavones, are known to be abundant in flower buds, while proanthocyanidins are found in higher amount in unripe fruits [29]. In hawthorn, using capillary zone electrophoresis, HPLC, and spectrophotometric analyses, the highest level of flavonoids (rutin, vitexin, vitexin-2′′-*O*-rhamnoside, and hyperoside) was registered in the leaves collected from the top branches of the trees [30]. In addition to the polyphenolic-based antioxidant agents, a number of other compounds have been found, which may contribute to the nutritional value and medicinal properties of the plant. For example, the flowers contain high levels of tocopherols, ascorbic acid, and show a good *n*-6/*n*-3 fatty acid ratio, in comparison with ripened fruits, while unripe fruits generally contain the highest level of polyunsaturated fatty acids [11].

In the following paragraphs, the main *C. monogyna* chemical classes of biological significance will be systematically presented.

### 4.1. Flavonoids

Flavonoids are a class of polyphenolic compounds that are ubiquitously distributed in the plant kingdom. Structurally, they are composed of two six-member aromatic rings (A and B) joined together by a three-carbon chain that may cyclize to form the third ring, C (1). Depending on the position of the B-ring bond on the three-carbon linking chain (carbon-2, 3, or 4 position) or the chemistry of the linking chain (e.g., presence/absence of a double bond, cyclization, presence/absence of a ketone functional group, *etc.*), flavonoids are further subdivided into several classes, such as flavones, flavonols, flavanones, flavans, anthocyanidins, isoflavones, neoflavones, and chalcones. Due to their numerous pharmacological activities, ranging from antioxidant capacity to protective activity against chronic disease, such as cancer, diabetes, and inflammation, flavonoids are by far the most studied classes of secondary plant metabolites [31,32,33,34]. The chemical structure of the flavonoids occurring in *C. monogyna* are reported in Figure 1.

#### 4.1.1. Flavan-3-ols

**Figure 2:**
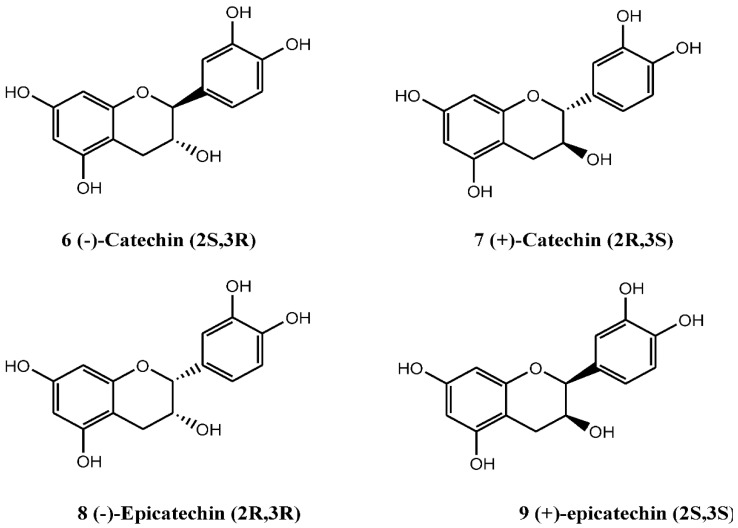
The flavan-3-ol catechin compounds of *C. monogyna.*

The flavan-3-ol compounds, containing diorthohydroxyl (catechol) function group at ring-B, are among the most common flavonoids known to date. Based on the C-2 and C-3 chiral centers, four stereoisomers, (±)-catechins, and (±)-epicatechins (Figure 2, compound numbers 6, 7, 8, 9), are possible. Interestingly, all these forms, either by their own or as part of complex structural components, have been found in *C. monogyna*. As a monomer, (−)-epicatechin appears to be abundantly present in the plant, while (+)-catechin is a minor component found both in the aerial parts and cell suspension cultures [13,35].

#### 4.1.2. Procyanidins

**Figure 3:**
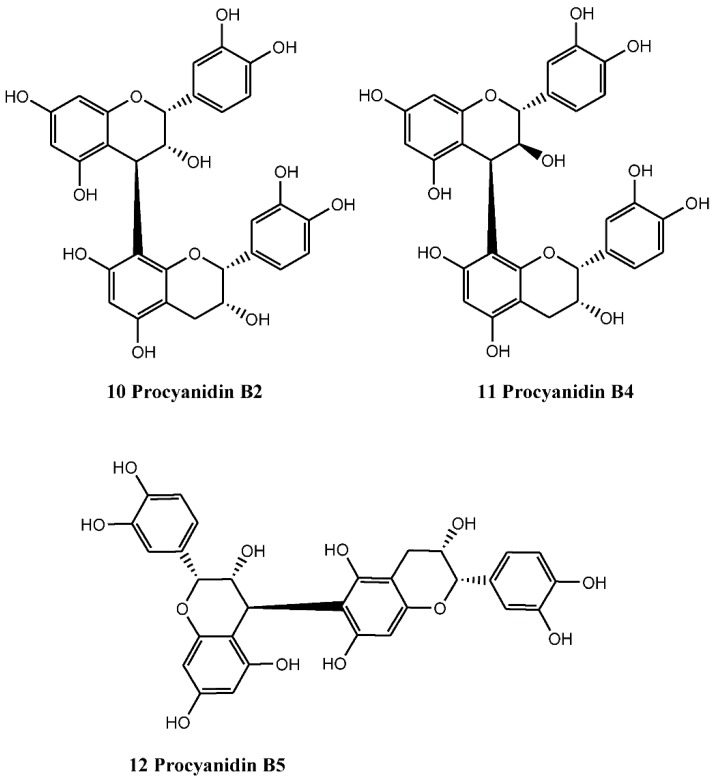
Dimeric catechins (procyanidins) isolated from various parts of *C. monogyna.*

**Figure 4:**
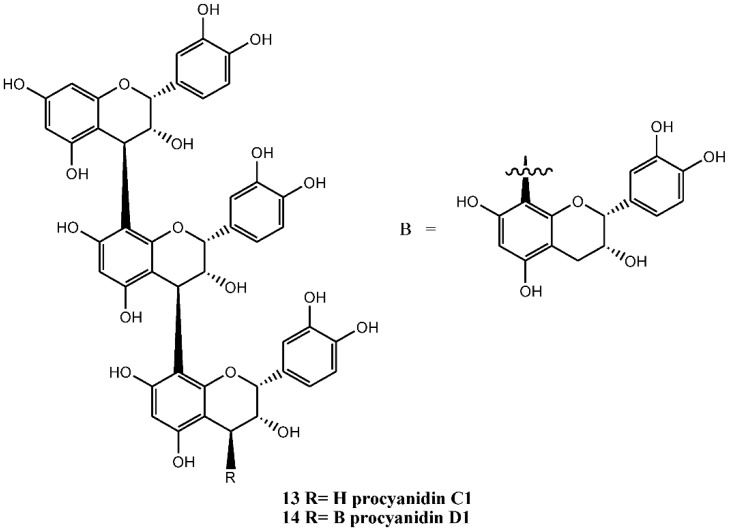
The (−)-epicatechin trimeric (C1, 13) and tetrameric (D1) of *C. monogyna.*

Catechins and epicatechins often undergo oxidation reactions in plants to form dimers, trimmers, and oligomeric structures, called procyanidins. These macromolecules are good examples of the larger proanthocyanidin, or condensed tannins, formed by the condensation of flavans. The list of dimeric catechins (procyanidins) isolated from various parts of *C. monogyna* include B2 (compound number 10), B4 (compound number 11), and B5 (compound number 12) (Figure 3), and other catechin combinations that could possibly be present. The (−)-epicatechin trimeric (C1, compound number 13) and tetrameric (D1, compound number 14) have also been identified in the plant. To date, the identified procyanidins (compound numbers 10, 11, 12, 13, 14) (Figure 4) belong exclusively to B-type group, in which monomers are linked through single C-4/C-8 or C-4/C-6 interflavanol linkages. These compounds are also found in cell suspension cultures and are mainly composed of (−)-epicatechin units as a major component and (+)-catechin as a minor component [13,36].

#### 4.1.3. Flavones and Flavonols

**Figure 5:**
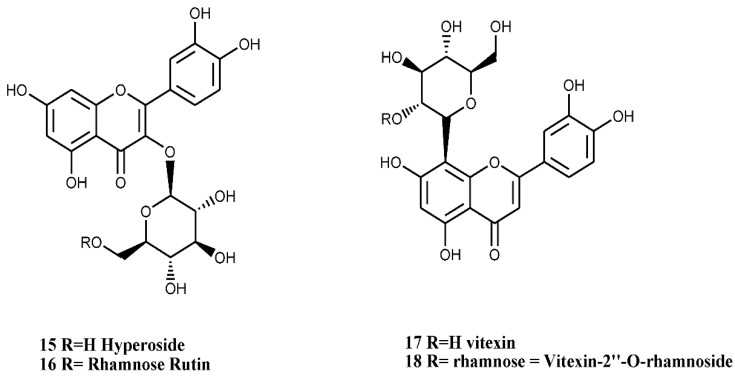
Isolated flavonols with catecholic moiety at ring-B.

**Figure 6:**
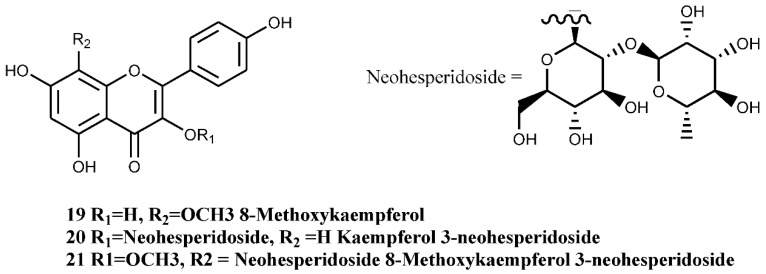
8-Methoxykaempferol 3-neohesperidoside and other flavonoids of the bee pollen of *C. monogyna*.

**Figure 7:**
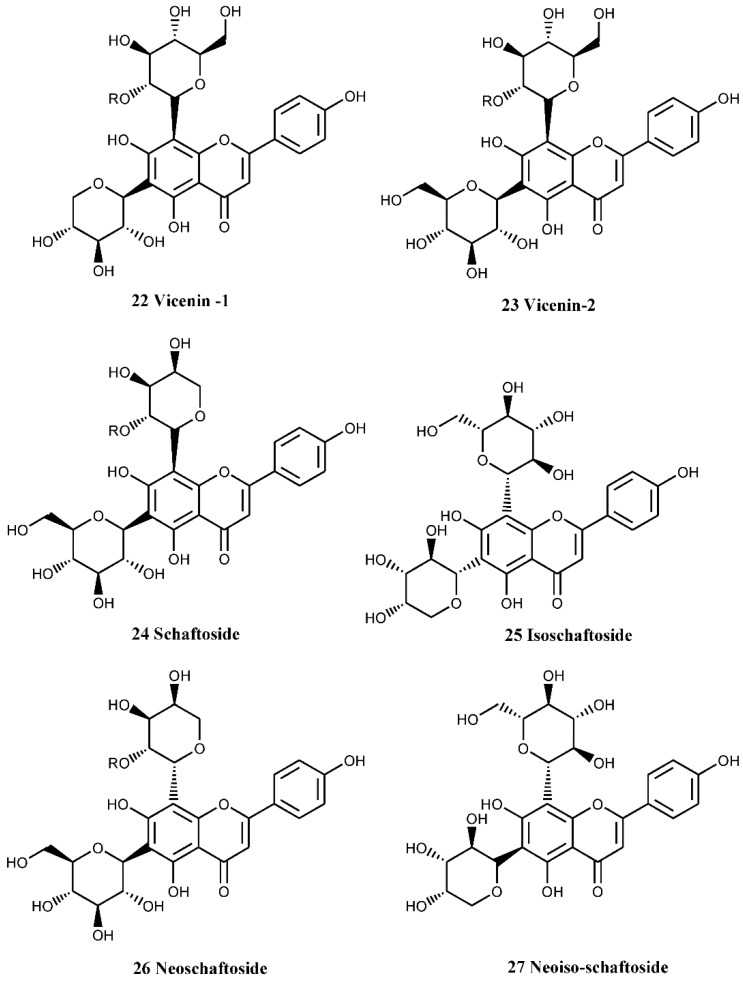
Analogues of di-C-glycosylapigenins of *C. monogyna* leaves.

In *C. monogyna,* a number of flavones and flavonols with catecholic moiety at ring-B have been isolated. Quercetin-3-*O*-glucoside (hyperoside, compound number 15, Figure 5), a flavonol glucoside, has been isolated from the callus cultures of the plant [35]. Hyperoside is also known to be one of the major components of the flowers [36]. In addition to this compound, a rare C-glycosylated flavone derivative, linked to glucose and rhamnose (Figure 5, compound numbers 16, 18), has also been isolated from the leaves [37]. 8-Methoxykaempferol 3-neohesperidoside and other flavonoids (Figure 6, compound numbers 19, 20, 21) have been isolated from the bee pollen of *C. monogyna*, while Nikolov *et al.* [37] have identified six analogues of di-*C*-glycosylapigenins (Figure 7, compounds numbers 22, 23, 24, 25, 26, 27) from the leaves.

#### 4.1.4. Anthocyanin and Anthocyanidins

**Figure 8:**
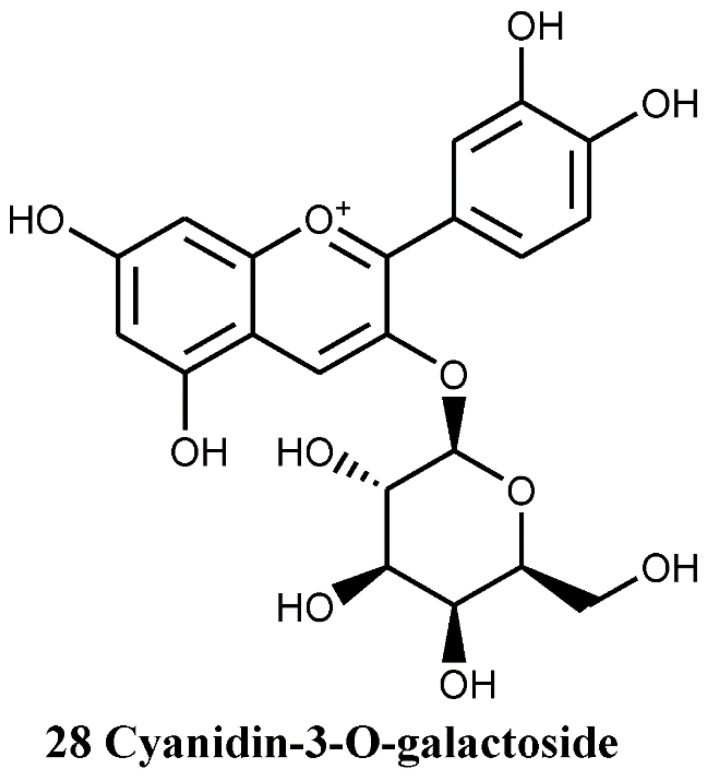
Flower pigment of *C. monogyna.*

Anthocyanidin glycosides (anthocyanins) are the main constituents of flowers, giving them their distinctive colour. Therefore, the identification of cyanidin-3-*O*-galactoside (Figure 8, compound number 28) as a flower pigment of *C. monogyna* by Froehlicher *et al.* [38] is not surprising.

### 4.2. Chlorogenic Acids

**Figure 9:**
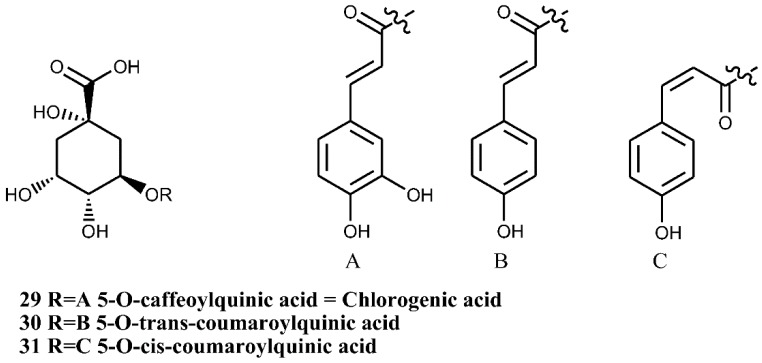
Chlorogenic acid and its isomers from flowers and cell suspension cultures of *C. monogyna*.

Chlorogenic acid and its isomers (Figure 9, compound numbers 29, 30, 31) are the major components of the flowers [38,39] and cell suspension cultures of *C. monogyna* [35]. An investigation on the flower composition, using HPLC–DAD–ESI/MS analysis, has indicated the presence of other caffeoylquinic acids, including 3- and 4-*O*-caffeoyl derivatives. Nevertheless, the presence of these compounds was not confirmed through isolation [36].

### 4.3. Triterpenes

**Figure 10:**
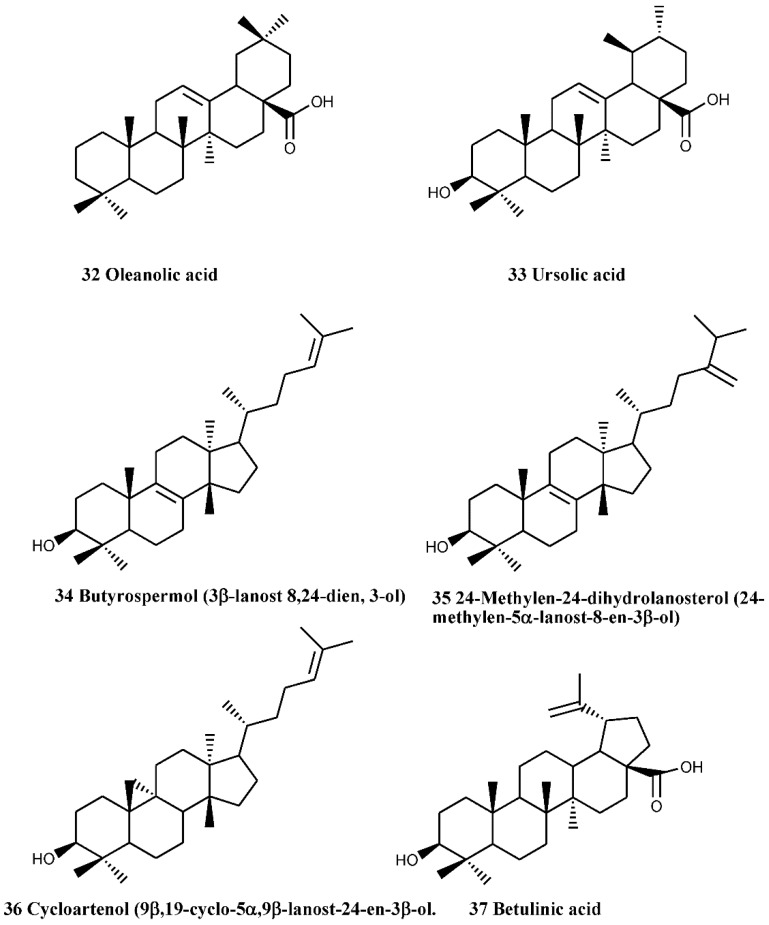
Triterpenes of the aerial parts (including twigs, stems and leaves) of *C. monogyna.*

By using GC-MS method, Caligiani *et al.* [40] have identified betulinic (compound number 37), oleanolic (compound number 32) and ursolic acids (compound number 33) in the flower extracts of *C. monogyna*. Butyrospermol (compound number 34), 24-methylen-24-dihydrolanosterol (compound number 35) and cycloartenol (compound number 36) along with simple aliphatic alcohols have also been isolated from the aerial parts (including twigs, stems and leaves) of the plant (Figure 10) [41].

## 5. Biological and Pharmacological Effects of *C. monogyna* Extracts

*C. monogyna* has a long history as medicinal plant used to treat kidney stones, digestive ailments, dyspnea and cardiovascular disorders. In Europe, hawthorn was first documented as a treatment for cardiovascular diseases in the late 1800s. Today, *C. monogyna* is used primarily to treat cardiovascular conditions due to its ability to reduce important risk factors such as inflammation, hypertension, and thrombosis [42]. Literature search shows that there is substantial evidence supporting the use of hawthorn in chronic congestive heart failure [43]. Even if Chinese hawthorn (*C. pinnatifida*) is the most studied [44,45,46,47] in the literature, we can also find some pharmacologic studies where *C. monogyna* has been studied in *in vitro* and *ex vivo* conditions, and in animal and human studies [43].

### 5.1. Cardiovascular Effects Registered in in Vitro and ex Vivo Experiments and in Animal Model Systems

In 2006, an *in vitro* study by Long *et al.* [48] showed that several hawthorn preparations have negative chronotropic effects in a cultured neonatal murine cardiomyocyte assay using unpaced cells. The hawthorn effects resulted to be different from those registered with conventional cardioactive drugs such as epinephrine; milrinone; ouabain; and similar to that registered for propranolol; regarding its negative chronotropic activity. Nevertheless; while propanol induced arrhythmia in the majority of the treated cardiomyocytes; on the contrary; hawthorn extract improved rhythmicity. Moreover, in the same research aimed at the evaluation of hawthorn extract mechanism of action; the authors showed that the chronotropic mechanism of action is not due to beta-adrenergic receptor blockade [48]. More recently, the same authors published the results of further investigation showing that hawthorn extract decreased the contraction rate of cultured cardiomyocytes via muscarinic receptor activation [16].

A triterpene fraction, isolated from the hexane extract and containing cycloartenol as the main component (80.87%), was tested for its anti-inflammatory activity in experimental animals in which inflammation was induced by carrageenan. In rats, at the highest oral dose (40 mg·kg^−1^), the hind-paw edema inhibition was 61.5% and 52.5% at 3 and 5 h of treatment, respectively. At the doses of 10, 20, and 40 mg·kg^−1^, peritoneal leucocyte infiltration inhibition was 41.9%, 64.7%, and 89.4%, respectively. The same triterpene fraction was also submitted to an *in vitro* test to verify its capacity to inhibit phospholipase A_2_. The results showed that it had weak inhibitory capacity. These results suggest that this *C. monogyna* fraction exerts *in vivo* anti-inflammatory activity [49].

The effects of the main flavonoids occurring in hawthorn, at concentrations ranging from 10^−7^ to 5 × 10^−4^ mol/L, were tested in Langendorff perfused isolated guinea pig hearts. At the highest tested concentration (0.5 mmol/L), *O*-glycosides luteolin-7-glucoside, hyperoside, and rutin increased the coronary flow (by about 186%, 66%, and 66%, respectively), and the relaxation velocity (by about 104%, 62%, and 73%, respectively), suggesting a potential health benefit on cardiac functions [50].

More recently, Attard *et al.* showed the inhibitory activity of the hydroethanolic extract against angiotensin-converting enzyme (ACE) [51]. The study of the chemical composition of this extract showed the presence of triterpenic acids, flavonoids and coumarins. The hydroethanolic extract, oleanolic acid (one of the main components of the extract), and captopril (used as positive control) showed IC values of 335.00 μg/mL, 3.61 μM, and 46.9 nM, respectively. These results indicated the anti-ACE activity of oleanolic acid extracted from *C. monogyna*. In 2013, Ferrugia *et al.* confirmed these results showing that terpenes possess *in silico* predicted ligand binding affinities to ACE receptors higher than that registered for captopril, enalaprilat and lisinopril [52].

Thrombosis is another important mechanism of development of cardiovascular disease. The ethanolic extract obtained from *C. monogyna* leaves was investigated for its anti-thrombotic effect in an animal model system in which tail thrombosis was induced by carrageenan. At the doses of 200 and 300 mg/kg, the extract resulted to be able to reduce the lengths of tail thrombosis in comparison to heparin, used as a positive control. The anti-thrombotic effect decreased after 48 and 72 h, nevertheless, the registered decrease was still significant after 72 h when the highest dose (300 mg/kg) was administered. The authors conclude that *C. monogyna* ethanolic extract could be used as a therapeutic agent or complementary treatment against thrombosis [53].

### 5.2. Effect on the Nervous System

In the Mongolian gerbil stroke model, hawthorn flavonoids decreased reactive oxygen species production in brain homogenates in a dose-dependent manner [54]; moreover, they reduced the inflammatory cytokines levels and showed protective effects in the ischemia/reperfusion injury model [55]. Hawthorn is known to protect the brain against ischemia-reperfusion and to improve behavior [56]. The underlying mechanism was attributed to the reduction in lipid peroxidation and nitric oxide levels by decreasing peroxynitrite formation [56]. On the other hand, hawthorn pulp and seed extracts caused central nervous system (CNS) depression and decreased exploratory behavior and locomotor activity in experimental animals [57]. In addition, hawthorn also showed analgesic effects, which were antagonized by naloxone, suggesting that the opioid receptors mediated analgesic effects [57].

### 5.3. Other Beneficial Therapeutic Effects of C. Monogyna

Hawthorn extract oral administration causes anti-inflammatory effect in carrageenan-induced rat paw edema model [39]. The same study reported that at the dose of 200 mg/kg, hawthorn extract shows 72.4% effectiveness, whereas indomethacin showed 50% reduction of rat paw edema at the given dose. Hawthorn is also reported to possess gastro-protective activity in a rat model of ethanol-induced acute stress ulcer, which resulted to be comparable to that of ranitidine, used as a positive control [39]. Moreover, the same study reported also the antimicrobial activity of the extract that showed moderate bactericidal activity, especially against Gram-positive bacteria such as *Micrococcus flavus*, *Bacillus subtilis*, and *Lysteria monocytogenes*, with no effect against *Candida albicans* [39]. Belkihir *et al.*, who studied the chemical composition of the phenolic extracts prepared from leaf, fruit, and syrup also investigated the antimicrobial activity of *C. monogyna* extracts. These extracts, which contain hyperoside and procyanidins as main compounds, showed high *in vitro* antioxidant and antiradical activity and weak antibacterial activity, especially against Gram-positive bacteria such as *Staphylococcus aureus* and *Streptococcus faecalis* [58].

*C. monogyna* extract was also investigated to identify potential migraine therapeutic agents together with other vegetable extracts, obtained from eighteen plants that were screened to detect plant constituents affecting ADP induced platelet aggregation and [14C]5-hydroxytryptamine release. In *in vitro* conditions *C. monogyna* extract inhibited ADP induced human platelet [14C]5-hydroxytryptamine release and caused significant inhibition of ADP induced platelet aggregation. The authors conclude that further studies elucidating the compounds responsible for these anti-platelet effects are needed to determine their exact mechanism of action [59].

Due to the high antioxidant activity, *C. monogyna* leaf hydroalcoholic extract has been exploited as an ingredient of innovative pharmaceutical formulations, such as hydrosoluble gels. Several semisolid formulations were prepared with different hawthorn parts extracts and the hydrosoluble gels, which presented good physico-chemical properties (consistency, color, and texture) and kept the antioxidant activity exhibited by the extracts from which they were prepared. These results suggested that *C. monogyna* extract is a good ingredient for potential dermopharmaceutical products [60].

### 5.4. Therapeutic Effects of Drug-Derived Toxicity

Treatment of various toxicities is an important ongoing issue for humankind. Many drugs cause various types of toxicities, and in particular antineoplastic drugs can cause multiple toxicities. Therefore, the treatment of drug-derived toxicity is an important ongoing issue for humankind. Cyclophosphamide is extensively used as an anti-neoplastic agent and possesses potential to cause testicular toxicity [61]. *C. monogyna* fruit extract has been found to reduce toxicity and improve testes and epididymis weights, along with improvement in the spermatogenic activity *C. monogyna* improved cyclosporine-induced reproductive toxicity: following treatment with fruit extract, an increase in sperm count and decrease in DNA damage in sperm cells have been registered [62]. *C. monogyna* has also shown promising results against doxorubicin toxicities, including reproductive toxicity, rescuing sperm count, and motility [63]. Hawthorn possesses the ability to reduce the oxidative stress and genotoxicity caused by the cyclophosphamide in mouse bone marrow cells [64]. Treatment of human subjects with hawthorn extract protects the isolated blood lymphocytes against methyl methanesulfonate genotoxicity and reports less binucleated cells (36% protection) in extracted and treated lymphocytes [65]. Similarly, lymphocytes isolated from hawthorn-treated human subjects, resulted to be protected against gamma irradiations (cobalt-60 γ irradiation) [66]. These studies suggest the huge potential of hawthorn in the treatment of various toxicities.

## 6. Adverse Effects/Toxicity of *C. Monogyna*

Considering its culinary uses, it can be hypothesized that hawthorn causes negligible adverse effects [67]. At therapeutic dosages, hawthorn causes very limited adverse effects, such as sweating, headache, mild rash, palpitations, sleepiness, agitation, and gastrointestinal adverse effects [42]. A systemic review analyzing 5577 patients, to whom standardized hawthorn extracts were administered, showed that most of the adverse effects ranged from mild to moderate [67]. Up to now, clinical studies have shown that there are no significant adverse effects associated with hawthorn consumption [67]. The median lethal dose (LD_50_) for oral administration of hydroalcoholic extract of leaf and fruits of hawthorn is 18.5 mL/kg in mice and 33.8 mL/kg in rats. In addition, it has been reported that median lethal dose (LD_50_) for intravenously-administered flavonoid-rich fractions of hawthorn is 1.56 g/kg in mice [9]. However, median lethal dose (LD_50_) for proanthocyanidins fraction is 130 mg/kg (intraperitoneal injection) and 300 mg/kg (subcutaneous injection) in mice [68].

## 7. Drug Interactions

Hawthorn may have the potential to interact with vasodilator drugs. In fact, hawthorn may potentiate or inhibit the actions of drugs used for hypertension, angina, heart failure, and arrhythmias [42]. In addition, hawthorn consumption may have some interactions with drugs, such as beta-blockers, digitalis, and some hypotensive agents, due to its cardiotonic and hypotensive effects [68].

## 8. Clinical Impact of *C. Monogyna*

Due to its minimal adverse effects, its *in vitro* biological properties, and its pharmacological activities registered in experimental animals, hawthorn can be used as therapeutic agent for the treatment of various human diseases. In clinical trials, the most studied hawthorn extracts are WS 1442 and LI 132. WS 1442 (called Crataegutt) is a leaf and flower extract that has a high content of procyanidins (standardized to 18.75%). LI 132 (called Faros) is prepared from the leaves, flowers, and berries and is standardized to 2.2% flavonoids.

The first studies on WS 1442 go back to the 1990s. More recently, Holubarsch *et al.* in 2000 published the first paper on the SPICE project, which is the first, international, randomized, placebo-controlled, double-blind study, aimed to investigate the effect Crataegus Special Extract WS 1442 on mortality of patients suffering from congestive heart failure [69]. In 2008 [70] the same research group published the results obtained from this multicenter study, which involved 2681 adults, with NYHA class II or III CHF and reduced left ventricular ejection fraction (LVEF< or =35%), receiving 900 mg/day WS 1442 or placebo for 24 months (WS 1442: 1338; placebo: 1343). The results showed that WS 1442 had no significant effect on the average time to first cardiac event and on cardiac mortality reduction. Nevertheless, WS 1442 reduced sudden cardiac death by 39.7% in the treated group in comparison with placebo group in those patients with less compromised left ventricular function.

In 2001, Zapfe published a randomized, placebo-controlled, double-blind clinical study on the efficacy and safety of Crataegus extract WS 1442 (standardized to 18.75% oligomeric procyanidines), performed on 40 female and male outpatients with congestive heart failure NYHA class II, treated for 12 weeks with either WS 1442 (3 × 1 capsule) or placebo. The registered laboratory parameters and adverse events showed that WS 1442 was safe and well tolerated. The outcomes were exercise tolerance and difference of the double product (heart rate × systolic blood pressure × 10^−2^). As regards the first outcome, the results showed that there was an increase of about 11% in the exercise tolerance (determined with bicycle exercise testing) in the WS 1442 group and a decrease of about 17% in the placebo group. Moreover, regarding the difference of the double product, a decrease of 27% in the WS 1442 group and 2.7% in the placebo group, were registered. In the whole, the data showed Crataegus extract WS 1442 is active in patients with congestive heart failure corresponding to NYHA class II [71].

In 2003, Degenring *et al.* showed that another registered Crataegus extract obtained from berries (Crataegisan^®^) resulted to be active and safe against patients with cardiac failure NYHA class II. In fact, the treatment with Crataegisan^®^ improved the exercise tolerance, without adverse effects, suggesting that in NYHA II patients there is an improvement in heart failure conditions under long-term therapy with this standardized extract [72].

In the last decade, other similar clinical studies were performed in which the safety and moderate beneficial effects in the treatment of chronic heart failure (New York Heart Association classes I to III) were described [73,74]. These clinical studies prompted Cochrane to publish a systematic review to define benefits and harms of hawthorn leaf and flower extract for treating patients with chronic heart failure. As regards the selection criteria, the included studies were randomized, double-blind, and placebo-controlled. The results of selection showed that fourteen trials met the inclusion criteria, but due to the fact that they did not all measure the same outcomes and several studies did not explain what kind of treatments patients were receiving, the clinical trials used for meta-analysis were ten studies, including 855 patients with chronic heart failure. These trials showed improvements in heart failure symptoms and in the function of the heart. The reported adverse events (*i.e.*, nausea, dizziness, and cardiac and gastrointestinal complaints) were mild, sporadic and transitory. The conclusion of this meta-analysis confirm the positive effect of hawthorn extract used in addition to conventional treatments for chronic heart failure [75].

Moreover, a search on the ClinicalTrials.gov database [18], with keywords “hawthorn” and “*Crataegus monogyna*” has shown that there are 23 clinical trials on this plant, of which 11 have been completed. Our search has also found that there are further four recruited clinical trials and five not yet recruited ones, while three clinical trials are of unknown status. Table 1 contains titles of completed clinical trials, their references numbers, study type, as well as disease conditions.

## 9. Conclusions

In conclusion, hawthorn is both a medicinal plant, which is used in folk medicine, and a common edible plant, which is widely used for the preparation of different foodstuff. Thus, based on the common use as traditional medicine and food, *C. monogyna* can be considered safe. Moreover, clinical trials showed no significant adverse effects. The present review has shown that hawthorn possesses a range of pharmacological effects, due to the presence of various bioactive natural compounds, such as flavanonoids and triterpenic compounds. In particular, hawthorn can be used in the prevention and/or mitigation of cardiovascular diseases. In fact, it is able to reduce cardiovascular risk factors, such as hypertension, thrombosis, *etc.*, and has beneficial effects on cardiac functions. Therefore, due to its multiple health-promoting effects, hawthorn can be recommended for future clinical trials aimed at examining its beneficial effects. In addition, we recommend that future research on *C. monogyna* should focus on:

(1) finding the best cultivation protocols and a way for increasing its production;
(2) finding the bioactive constituents, which are the most responsible for its pharmacological effects and, thereafter, increasing its production through biotechnological protocols;
(3) increasing the bioavailability of its bioactive constituents;
(4) ascertaining the most effective dose for its clinical efficacy;
(5) finding the exact molecular mechanisms responsible for its pharmacological effects.
